# Quantifying lion (*Panthera leo*) demographic response following a three-year moratorium on trophy hunting

**DOI:** 10.1371/journal.pone.0197030

**Published:** 2018-05-21

**Authors:** Thandiwe Mweetwa, David Christianson, Matt Becker, Scott Creel, Elias Rosenblatt, Johnathan Merkle, Egil Dröge, Henry Mwape, Jones Masonde, Twakundine Simpamba

**Affiliations:** 1 Zambian Carnivore Programme, Eastern Province, Mfuwe, Zambia; 2 School of Natural Resources and the Environment, University of Arizona, Tucson, Arizona, United States of America; 3 Department of Ecology, Montana State University, Bozeman, Montana, United States of America; 4 Rubenstein School of Environment and Natural Resources, University of Vermont, Burlington, Vermont, United States of America; 5 Wildlife Conservation Research Unit, University of Oxford, Tubney, United Kingdom; 6 Department of National Parks and Wildlife, Chilanga, Zambia; Université de Sherbrooke, CANADA

## Abstract

Factors that limit African lion populations are manifold and well-recognized, but their relative demographic effects remain poorly understood, particularly trophy hunting near protected areas. We identified and monitored 386 individual lions within and around South Luangwa National Park, Zambia, for five years (2008–2012) with trophy hunting and for three additional years (2013–2015) during a hunting moratorium. We used these data with mark-resight models to estimate the effects of hunting on lion survival, recruitment, and abundance. The best survival models, accounting for imperfect detection, revealed strong positive effects of the moratorium, with survival increasing by 17.1 and 14.0 percentage points in subadult and adult males, respectively. Smaller effects on adult female survival and positive effects on cub survival were also detected. The sex-ratio of cubs shifted from unbiased during trophy-hunting to female-biased during the moratorium. Closed mark-recapture models revealed a large increase in lion abundance during the hunting moratorium, from 116 lions in 2012 immediately preceding the moratorium to 209 lions in the last year of the moratorium. More cubs were produced each year of the moratorium than in any year with trophy hunting. Lion demographics shifted from a male-depleted population consisting mostly of adult (≥4 years) females to a younger population with more (>29%) adult males. These data show that the three-year moratorium was effective at growing the Luangwa lion population and increasing the number of adult males. The results suggest that moratoria may be an effective tool for improving the sustainability of lion trophy hunting, particularly where systematic monitoring, conservative quotas, and age-based harvesting are difficult to enforce.

## Introduction

African lions *(Panthera leo)* are declining world-wide and this is a concern given their role in ecosystem function [1] and their economic value to phototourism and trophy hunting [2]. Lions are estimated to have lost over 90% of their historical range and total population estimates which, while debated, range between 20,000–32,000 [3] with range loss and population declines accelerating in many areas. At present, few countries possess strong evidence for stable lion populations [3–6]. In response to these concerning trends, the United States Fish and Wildlife Service (USFWS) uplisted lions in East and Southern Africa to threatened, and West and Central African populations to endangered [7].

Overwhelmingly, the primary threats to lions are anthropogenic, including habitat loss and human encroachment, conflict with humans and livestock, wire snare poaching, illegal hunting, disease, and prey depletion [8–12]. Most of these threats are similar in that they are typically unregulated or illegal, making them difficult to quantify and explicitly link to lion demography. In contrast, legal trophy hunting of lions is an anthropogenic impact that results in direct mortality of lions, but is unique because it is regulated and monitored and can confer both benefits and costs to conservation [13–15].

Because of the growing concern around the conservation status of the African lion, legal trophy hunting has become a controversial and polarizing issue [2, 16, 17]. There are significant financial benefits associated with lion hunting in that it is one of the most lucrative trophy species and can provide revenue to government and communities to incentivize protection of lion habitat and populations [14]. By targeting adult males, trophy-hunting is often argued to have inherently weak impacts on population growth because few males can breed with many females and because males often have lower survival than females, providing scope for compensatory hunting mortality. On the other hand, hunting of lions can disrupt social organization and cause superadditive effects on cub survival, and policies regulating hunting for lions and other large carnivores often fail to avoid negative population growth [15, 18]. Sustainable hunting is particularly important across the strong gradients of protection characterizing protected area networks where most trophy hunting in Africa occurs [15]. The impetus for the designation of protected areas often includes the creation of refugia where wildlife can be buffered from anthropogenic impacts, including trophy hunting. Thus, strictly protected areas and hunted areas are often interconnected parts of a larger conservation policy. Hunting areas often lie adjacent to protected areas where wildlife densities are high [19]. High turnover in male lions in hunted areas may cause males to leave protected areas to capitalize on adjacent pride male vacancies i.e., the vacuum effect [20]. Studies continue to demonstrate strong population impacts on lions from trophy hunting, ranging from male depletion, skewed sex ratios, elevated levels of infanticide, and population decline [12,15,20–28], but isolating the effects of trophy hunting from other sources of mortality, particularly in lion populations traversing gradients of protection has been difficult [13].

Zambia is one of the few countries with viable populations of lions although data on lion numbers are sparse save for 3 ecosystems, Luangwa, Mid-Zambezi, and Kafue, which comprise the country’s primary "Lion Conservation Units” [4]. The largest lion population is found in the Luangwa Valley in eastern Zambia and this area is considered one of the last 10 remaining strongholds for the species in Africa [4]. Rosenblatt et al. [27], using mark-recapture models fit to data from intensive monitoring of 210 individual lions in 18 prides and 14 male coalitions suggested that (superimposed on local conditions) trophy hunting was limiting this population. Hunting was the leading cause of death for males, with 46 males harvested. The median age of males harvested was 4.86 years old and annual harvests ranged from 1.86–2.56 lions/1000km^2^, in contrast to recommendations of a 6 year minimum age limit [29] and a harvest limit of 0.5 lions/1000km^2^ [24]. The population was declining with low recruitment, low subadult and adult male survival, and an adult female population shifted towards older age classes. Utilizing trophy data from harvested lions during this period, Creel et al. [15] found that males were shot a median distance of 900m from the park boundary, indicating strong source-sink dynamics between national park and Game Management Areas (GMAs) where hunting takes place. Concerns over population declines, poor recruitment, skewed sex ratios, and decreasing age of males harvested led the Government of Zambia through the Ministry of Tourism and Arts to enact a three-year moratorium on lion hunting from 2013–2015 to promote recovery of the population.

Here, we used observations of individually-recognizable lions collected 5 years before and 3 years during the moratorium on trophy hunting in a quasi-experiment to evaluate the impact of trophy hunting on lion survival, recruitment, and population size.

## Study area

**Fig 1:**
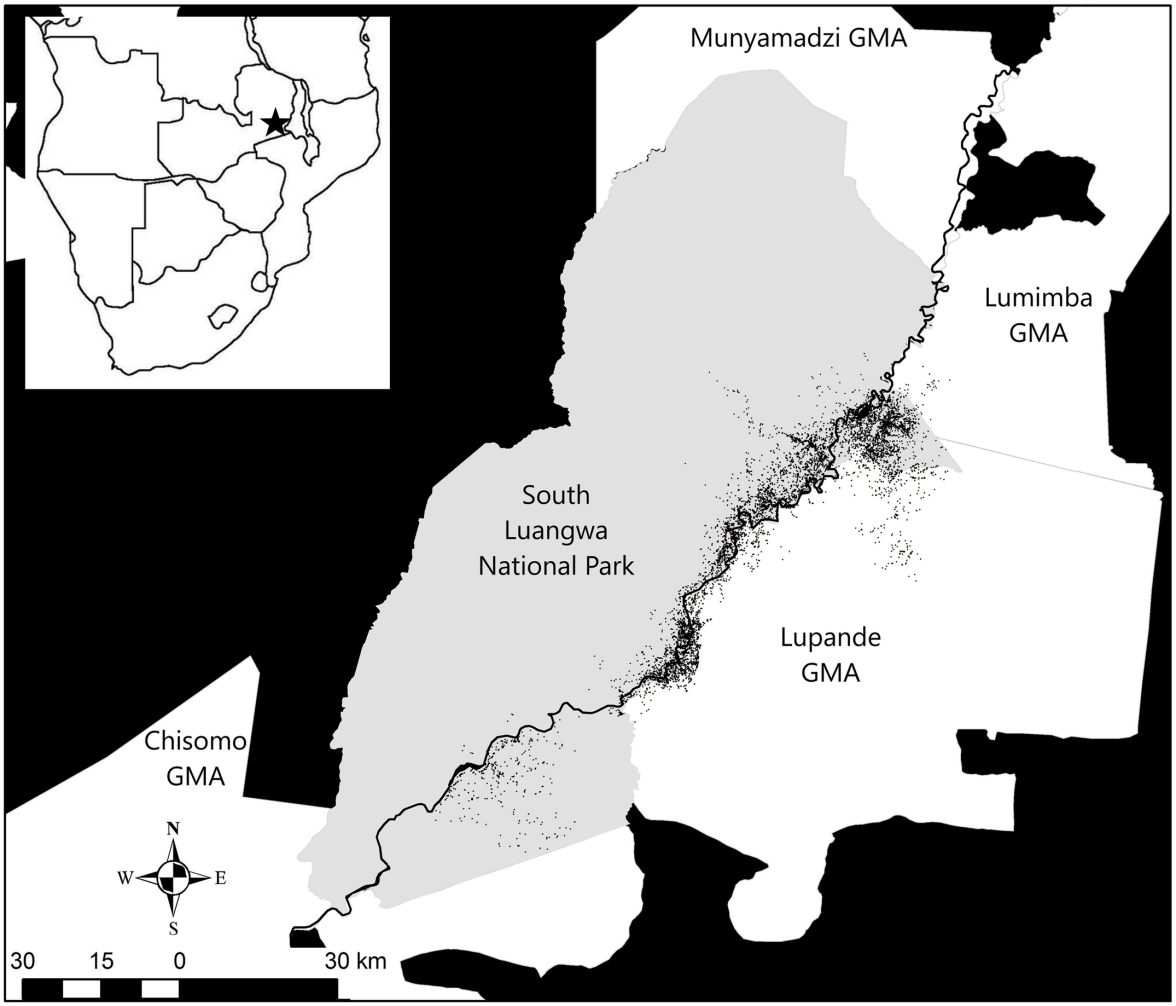
The study area in Eastern Zambia which formed the core area of use for lions monitored from 2008 to 2015. Adjacent to the national park are Game Management Areas (GMA—white) where trophy hunting of lions has been allowed, but were subject to a three-year moratorium on trophy hunting of lions from 2013 to 2015. Lion locations (points) from visual detections as well as radio-collar and GPS-collar telemetry lie primarily along the Luangwa river that flows from the northeast to southwest along the boundaries of the park and GMAs.

The 2775 km^2^ study area was located in the Luangwa river valley (31°50’E-32°5’E, 12°50’S-13°05’S) and consisted of the eastern side of South Luangwa National Park and the adjacent Lupande and Lumimba GMAs (Fig 1). The valley has elevation ranging from 500-800m, and experiences three main seasons; hot-wet (November-early April), cold-dry (May-July) and hot-dry (August-October). The most common vegetation types in the valley include Mopane woodlands (dominated by the species *Colophospermum mopane*), scrublands, open grasslands, and riparian woodlands [27]. The national park and adjacent GMAs hold high concentrations of ungulates. Settlement is prohibited in the national park and the main human land use is photographic safaris for tourists. The neighboring GMAs have mixed land uses, including photographic and hunting safaris. While some local residents are partially or fully dependent on the tourism industry, most are dependent on subsistence agriculture [30].

## Methods

Lion monitoring, individual identification, and detection have been described previously [27]. Briefly, we recorded all lion sightings from intensive monitoring of individually identifiable lions in 21 prides and 24 male coalitions from 2008 to 2015. Lions were individually identified using whisker-spot patterns, scarring, and tooth breakage, matching sighted lions or photographs against a catalog of known lions photographed from several angles [31]. At least one adult female lion was radio-collared in each of 19 resident prides for the duration of the study and one adult male lion in 4 resident male coalitions between 2009 and 2013, using a combination of VHF and GPS collars with permission and collaboration from the Department of National Parks and Wildlife (DNPW). Sightings and photographs from commercial guides, professional hunters, and tourists provided additional detections. Intensive monitoring was difficult during the wet season when most roads became impassable; thus we restricted sightings to the drier 8-month dry period (April–November) to avoid detection histories (see below) with periods of uniformly low detection.

We estimated the age of individuals first identified as adults using established standards for nose-pigmentation pattern, tooth wear and coloration, and facial scarring [29, 32, 33], validating the accuracy of standards against known-age individuals. We estimated the age of 1^st^ and 2^nd^ year cubs based on body size and the distinctness of spots in the pelage, especially on the belly. All individuals were initially assigned to age classes with a temporal resolution of 6 months. Most (226 out of 386) known individuals were first detected as first or second year cubs when age estimate was most precise [29, 32, 33]. Two females detected only once did not have their age estimated in the field, and were assigned the median age for (non-cub) females of 4.7 years. Forty-two cubs (18.7% of all cubs) could not be sexed before they disappeared from the observed lion population and were presumed dead. We arbitrarily assigned a gender to each of these cubs assuming a 50:50 sex ratio [34] using a rule based on ID number (even numbers = male, odd numbers = female). When modeling the effects of gender on survival (see below), we switched the assigned gender of these cubs and found no effect on our results.

### Modelling survival

We used detection records from a period of eight years (2008 to 2015) for 386 individually recognizable lions to fit Cormack-Jolly-Seber (CJS) models estimating annual survival and factors that affect it, while accounting for variation in individual detection. Rather than simply comparing the findings from Rosenblatt et al. [27] with results from the moratorium data, we combined all years into a single analysis to obtain greater power for assessing trophy hunting impacts. We initially compiled lion observations into monthly detection histories spanning April-November (8 month detection histories for each of 8 years) and then collapsed monthly detection histories into two-month occasions (4 occasions per year for 8 years or 32 total occasions) recording whether or not each individually recognizable lion was detected (1) or not (0) in each two-month occasion. In CJS, one model for survival and one model for detection are both fit simultaneously in hierarchical fashion but both processes can be influenced by an overlapping set of variables. Often model selection is used to compare models with unique combinations of parameters affecting both survival and detection [27]. Here, we used RMark and program MARK to resolve a best-supported CJS model in two separate stages of model selection. In the first stage, we resolved the factors influencing detection by fitting candidate CJS models using a single global model of survival with multiple candidate detection models, each candidate model exploring how individual traits and temporal heterogeneity affected detection. In the second stage, we compared CJS models with varying combinations of the effects of age-class, sex, and trophy hunting on lion survival but always using only a single detection model—the best-supported detection model from the first stage of model selection.

#### Estimating mean annual survival stage 1: Identifying parameters affecting detection probability

We first fit 46 candidate models that compared the effects of age-class, sex, season [cold-dry (Apr-Sept) or hot-dry (Oct-Nov)], and year on detection (*p*). We also considered models that allowed for overdispersion arising from individual heterogeneity in detection [35] by modelling a discrete mixture of individuals with 2 categories of detection (*p*_*low*_ and *p*_*high*_) with probabilities of belonging to each detection category π and 1-π, respectively. Pledger et al. [35] point out that when individual and temporal heterogeneity in effort is present (as was the case here), time effects on detection should be fit, otherwise individuals captured during sessions with more effective search effort will affect the estimation of the mixing parameter. Consequently, we compared candidate models that include a temporal effect of study year, season, or both. Our candidate list of detection models consisted of: three models including single temporal effects of season (Apr-Sept, Oct-Nov), study year (2008 to 2015), or the individual effects of gender (2 classes), two models that included the temporal effects of year and season (with and without an interaction), one model that included the effects of year and sex, one model that included the effects of season and sex, four models that included the effects of age and sex as specified in Rosenblatt et al. [27] with 5 age classes for females, and 3 age classes for males with and without effects of year, season, or both year and season, one model that included the effects of age and sex with 5 age classes for both males and females, and one model with no variation in detection due to age, sex, season, or year (null model). Thus, our candidate list of detection models compared models that included lion covariates, temporal covariates, or both covariate types, affecting detection. The effects of mixture class on detection was included by itself (one model) and in each of the above models as a singular additive term (13 additional models) and also as an interaction between mixture class and season, sex, or year (19 models).

To facilitate comparison and selection of detection parameters in this first stage of analysis, we fit an identical set of terms affecting survival derived from a previous study in this population [27]. That is, we modelled the effects of three age classes on survival: cub (0–2 years old), sub adult (2–4), and adult (4+) alongside separate gender adjustments to the effects of each age class (modelled as the effect of being female) but with three gender:age terms for adult lions based on whether they were young (4–6), prime (6–8), or old (8+) adult female lions. We fit a single additive term for effects of trophy hunting on survival in male lions ≥2 years old to minimize the potential for misspecification of survival to influence selection of the best detection model.

#### Estimating mean annual survival stage 2: Identifying parameters affecting mean annual survival

In the second stage of survival model selection, we compared CJS models that differed in how sex, age, and hunting affected survival, fitting each model with the best-supported detection terms identified in the first stage of analysis, described above. This stage of modelling primarily served to refine the model of age and sex effects on survival identified by Rosenblatt et al (2014), which only examined data collected prior to the hunting moratorium. In particular, we sought to compare Rosenblatt et al.’s [27] base model and other age-sex models to models that also included hunting effects on survival in both targeted (males ≥2 years) and non-targeted (cubs and females) age-sex classes.

We fit 286 candidate models of survival using all 8 years of data from 386 individuals with 4 observations per year. Consequently, from 1 to 31 time intervals from each individual could potentially contribute information toward the estimation of survival and detection. We compared 22 models of age and sex-specific survival that included as few as 3 and as many as 7 different age classes with lower terminal bounds on age classes at 0, 1, 2, 4, 6, 8, and 10 years using common demarcations for age-classes thought to affect lion survival from the literature [10, 12, 15, 27, 29]. We considered variations on the top model in Rosenblatt et al. [27], which fit age-specific survival for 5 age classes in both male and female lions, by also including models with: cubs split into two separate age classes, [0,1) and [1,2); additional variation in survival in old, [8+), adult female lions; no difference in survival between male and female cubs; and constant or variable survival across adult, [4+) years, age classes in both male and female lions (S1 Table).

The moratorium on hunting lions was modelled as a temporal effect by treating all time intervals from April 2008 to October 2012 as ‘hunting’ intervals and all subsequent intervals as ‘moratorium’ intervals. The first moratorium interval began with the last capture session of 2012 as the hunting moratorium was in effect over the majority of this interval (November 2012 to April 2013). In addition to modelling only age and sex class effects on survival, we considered models that also included the effects of the hunting moratorium as an adjustment term to age and sex class effects, including models with a singular hunting effect on (1) all lions, (2) on all male lions, (3) on males ≥2 years, or (4) on adult (≥4 years) males as well as models with multiple, separate hunting effects on: (5) subadult and adult males; (6) cubs and lions ≥ 2 years; (7) cubs and male lions ≥ 2 years; (8) cubs, male lions ≥ 2 years, and female lions ≥ 2 years; (9) cubs, subadult males, and adult males; (10) cubs and adult males; (11) 1^st^ year cubs and male lions ≥ 2 years; (12) and 1^st^ year cubs and adult males. We considered hunting effects on age-sex classes other than adult males because in social, cooperatively hunting and breeding carnivores, hunting has been shown to have demographic impacts that go beyond direct offtake of the targeted age-sex class [18].

Thus, for each of the 22 models of age-sex class effects on survival (S1 Table), we fit 13 variations of the effects of hunting (this included one variant with no hunting effect) and used AICc scores to compare these 286 models to (1) identify which model of age-sex structure in lions best explained variation in survival, (2) determine whether models with an effect of hunting on survival were better supported by the data than models without hunting effects, and (3) identify which age-sex classes showed responses in survival to the moratorium and (4) quantify the magnitude of these responses. While our candidate model list of survival models was extensive, we note that these models considered only the effects of sex, age, and trophy hunting. Differences among models primarily arose from variation in the number of age classes, the start and endpoints of age-classes, and whether or not gender or trophy-hunting affected survival in these age classes. Consequently, most models performed only marginally better or worse than other models with little additional inferential power (see Results). A narrower set of models would have likely produced clearer demarcation between strong and weak models but our large candidate list was also developed to illustrate the uncertainty surrounding age-sex class effects when estimating demography in a large carnivore population.

### Estimating abundance

We used monthly capture-recapture histories to estimate lion abundance in each year, accounting for individual and temporal heterogeneity in detection using closed mark-resight models [36]. Using RMark and program MARK, we fit a Huggins model with individual heterogeneity in detection to estimate the probability of initial detection, subsequent detection, and population size. We excluded detection histories from 39 lions that were known to have died before modelling (or were cubs that were counted as part of a litter but disappeared before they could be individually identified) but added these individuals to the population estimate for the year that they died. We used the same detection parameters from the top survival model to also model for estimating population size.

To mimic the parameterization of the detection process from the top CJS model of survival, we constrained the estimation of detection when estimating abundance by assuming no variation between initial capture and recapture probabilities within each season (i.e., *p* = *c*). This constraint kept abundance estimation consistent with the CJS framework which conditions the likelihood on first capture, and is justified given repeated observations of the habituated animals in our study. We also fit models with the mixing parameter (π) for individual heterogeneity fixed at its model-averaged estimate from the top CJS survival models (see Results). Fixing π maintained consistency in model structure through time (as one abundance model was fit for each year of data), facilitating comparisons of abundance across years as recommended by Williams et al. [37]. This approach also assumed individual heterogeneity in the lion population was constant throughout the study (i.e. the probability that an individual lion belonged to each detection class was fixed throughout their lives). This same assumption is implicit in CJS models of survival assuming individual heterogeneity in detection [35].

## Results

### Lion population structure

We individually identified 386 lions from 21 prides and 24 male coalitions from 2008 to 2015 and the number of known lions observed in any single year ranged from a low of 76 in 2008 to a high of 198 lions in 2015. Forty-six lions were harvested by hunting concessions from 2008–2012. Due to challenges in collecting photographs of harvested lions from hunters, we could only confirm the identity of 5 of the 46 males harvested from 2008 to 2012, yet we also documented the disappearance of 46 males over the age of 3 years old from the study population from 2008–2012. During the five years with hunting, we documented deaths from other anthropogenic sources (2), disease (1), infanticide (3), injuries (4), and unknown causes (27), including cubs of known females and known litter size that were no longer seen with their mothers and adult males that were no longer seen after a harvest was reported from the area last seen. During the three years of the moratorium, we documented 17 mortalities, including poaching (1), infanticide (9), disease (1), injuries (2), and unknown sources (4).

**Fig 2:**
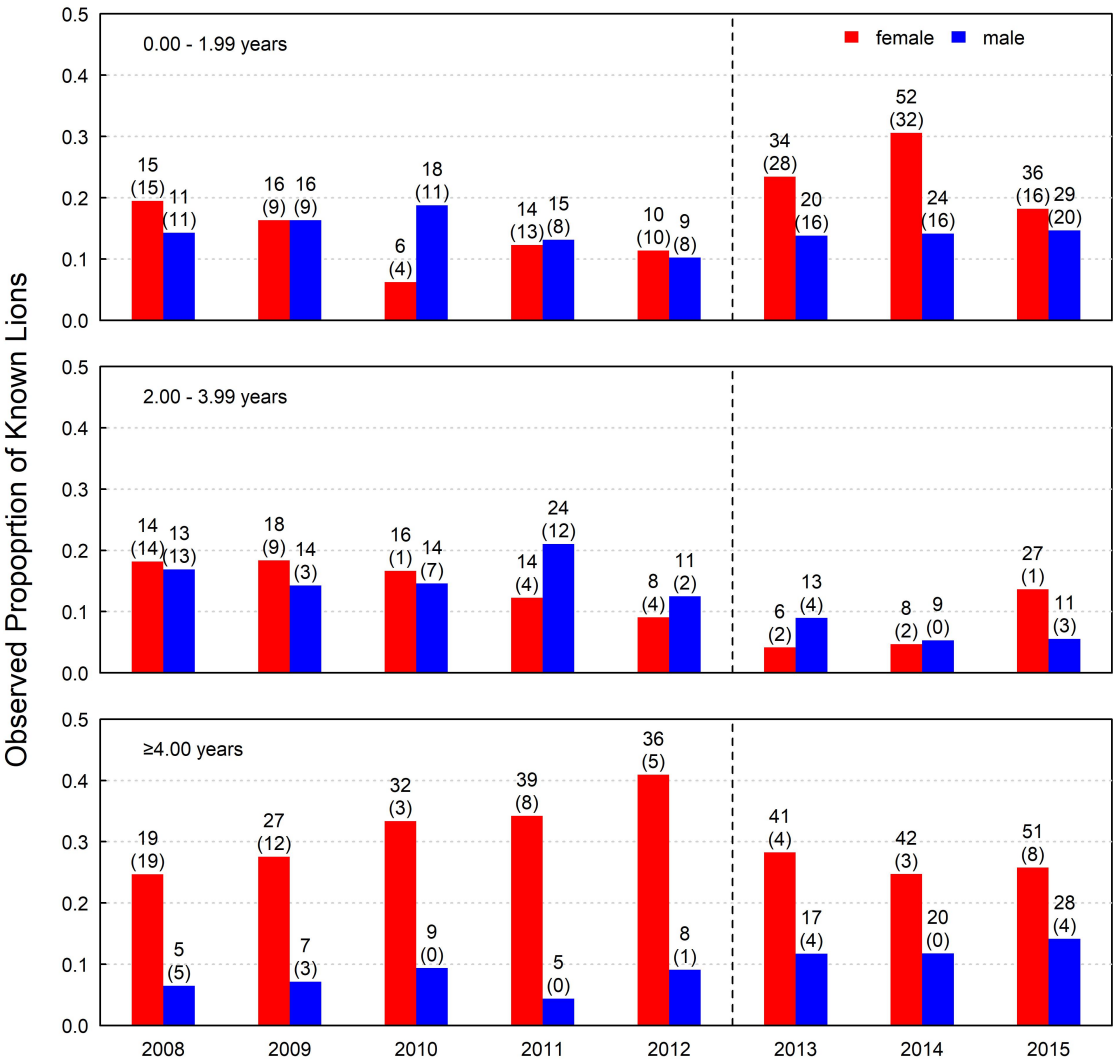
Observed proportion of male and female lions as cubs (0.00–1.99 years), subadults (2.00–3.99 years), and adults (≥4.00 years) in the South Luangwa lion population from 2008 to 2015. The vertical dashed line indicates when 3-year moratorium on trophy hunting was implemented. Numerals indicate known lions observed. Parenthetic numeral indicates known lions that were new to the study that year. These numbers include 43 cubs (18.7% of all cubs) that were observed, but not sexed before disappearance and were randomly assigned a gender (21 male and 22 female) for modelling survival.

Cubs were the most common age class of known lions observed (Fig 2). 33.7% of known lions were cubs in 2008 increasing to a high of 44.7% of known lions by 2014, indicating a shift towards a younger population during the moratorium. The mean number of litters of first-year cubs directly observed was 6.6 litters/year during the hunting period (range 6 to 7) but this more than doubled during the moratorium (15.0 litters/year, range 13 to 18). Litter sizes ranged from 1 to 5 cubs and did not differ between the hunting period and moratorium (2.61 cubs/litter vs. 2.64 cubs/litter, respectively, t = -0.149, P = 0.441. The sex ratio (females: males) of cubs was unbiased prior to the moratorium (95% CI: 0.69, 1.68, exact binomial test: P = 0.828), but was female biased during the moratorium (1.08, 2.55, P = 0.020). Adult males were rare, and decreased to an unusually low value of 4.4% of observed lions in 2011, one year before the hunting moratorium, before increasing to a high of 14.4% of observed known lions by 2015 (Fig 2). Observed adult males were considerably younger (6.5 ± 0.7 years, max: 11.9 years) than observed adult females (mean ± 95% CI: 8.9 ± 0.3 years, max: 13.9 years).

### Estimating mean annual survival stage 1: Parameters affecting detection probability

Over the 8 years following 386 individually recognizable lions noting whether they were observed or not every 2 months from April through November, we scored 2132 detections (5.52 detections/lion, range 1 to 29) for an effective sample size (omitting the last occasion where survival and detection become confounded) of 2002 for estimating detection. In our first stage of model selection to identify parameters affecting detection, all 32 models with individual heterogeneity in detection (modeled as 2 classes of detection, *p*_high_ and *p*_low_, with probability *pi* and 1-*pi*, respectively) performed better than any of the 13 models without individual heterogeneity. Models with variation in detection between years and seasons (along with individual heterogeneity in detection) were consistently better than models that modelled detection varying by age-sex classes. The top model was 4.65 AICc units better than the next best model and included the main effects of detection class, year, and season, plus all two-way and three-way interactions between these effects.

### Estimating mean annual survival stage 2: Parameters affecting mean annual survival

#### Effects of age and sex

**Fig 3:**
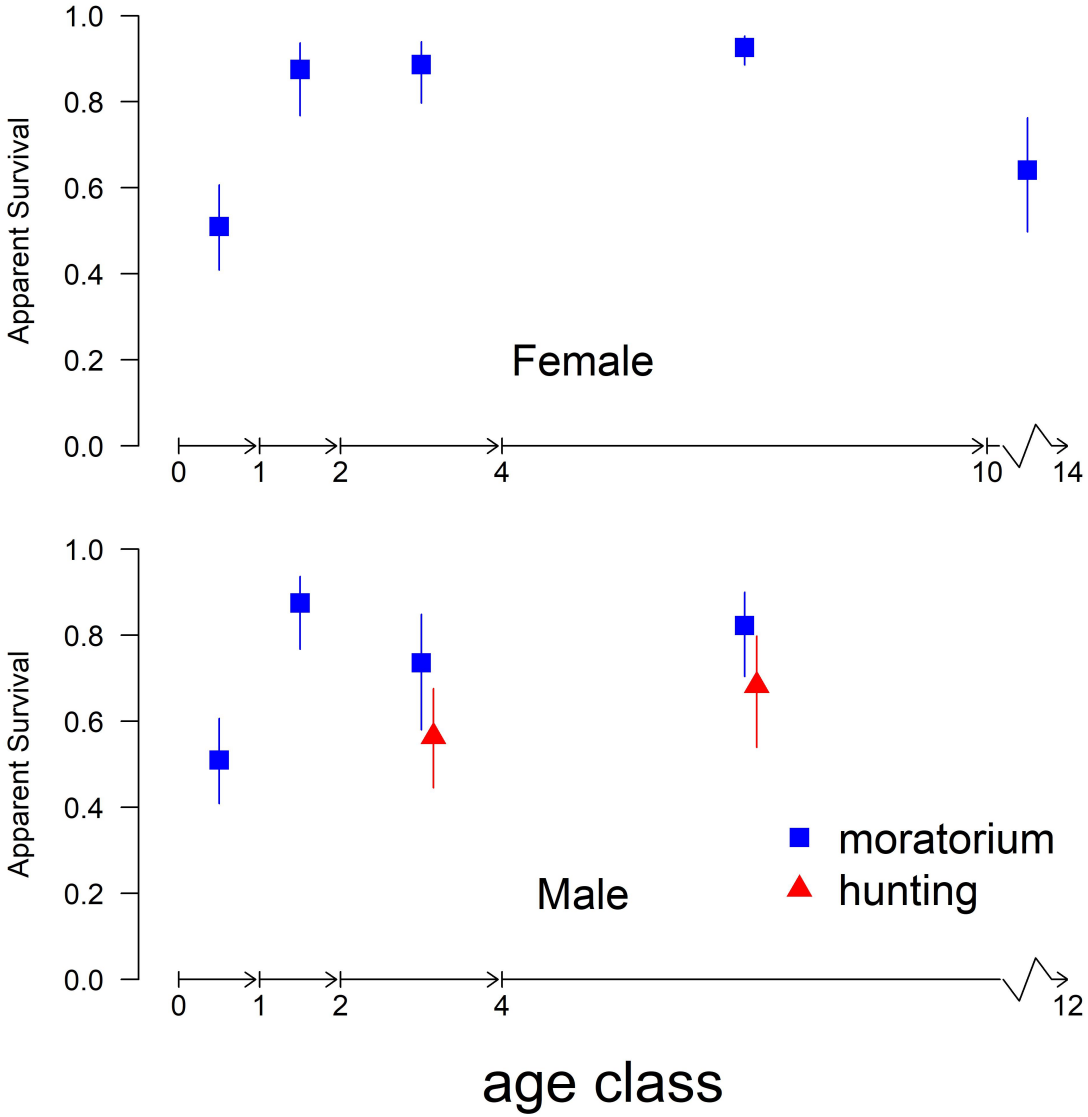
Apparent survival estimates (with 95% CI) in the South Luangwa lion population from the top Cormack-Jolly-Seber survival model. The oldest observed female and male lion determined the upper terminal bound in the oldest age class for each sex.

Three of 286 candidate survival models were within 2 AICc units of the top model (Table 1). Each of these four top models found lower survival in 1^st^ year cubs than in 2^nd^ year cubs, no difference in survival between male and female cubs (1^st^ year or 2^nd^ year), lower survival in males than in female lions for all age classes older than cubs, lower survival in subadult males than in either 2^nd^ year cub or adult males, no variation in adult male survival after age 4, and declining survival in adult females after age 10 (S2 Table). The top four models only disagreed as to whether a separate survival estimate in the 8–10 year-old age class in adult females was supported (3^rd^ best model, Table 1). Female survival was highest from age 4 to 10 at 0.93 (95% CI: 0.89, 0.95) and lowest in first-year cubs at 0.51 (0.41, 0.61) after exponentiation by 1.58 to account for mean age at first detection of 4.4 months in first year cubs. Survival in second year cubs was much higher at 0.87 (0.77, 0.94) and survival held steady for subadult females (0.88), but declined for subadult males even in the absence of hunting (Fig 3).

**Table 1:** The four best supported Cormack-Jolly-Seber models of survival as determined by AICc scores.

Model	k	Delta AICc	weights
{Phi(age_[0,1,2,4+)_ & ♀_[2,4,10+)_ & hunting:♂_[2+)_), p(mixture*season*year), pi(.)}	41	0.00	0.392
{Phi(age_[0,1,2,4+)_ & ♀_[2,4,10+)_ & hunting:age_[0,2)_ & hunting:♂_[2+)_), p(mixture*season*year), pi(.)}	42	1.25	0.210
{Phi(age_[0,1,2,4+)_ & ♀_[2,4,8,10+)_ & hunting:♂_[2+)_), p(mixture*season*year), pi(.)}	42	1.32	0.202
{Phi(age_[0,1,2,4+)_ & ♀_[2,4,10+)_ & hunting:age_[0,2)_ & hunting:age_[2+)_), p(mixture*season*year), pi(.)}	42	1.39	0.196

All 286 models (crossing 22 variants of age-sex structure with 13 different types of hunting effects, including no effect) were fit in the second stage of model selection using the terms affecting detection from the best model identified in the first stage of model selection. Factors affecting detection including individual heterogeneity modelled as a mixture (pi) of two classes, study year (2008–2015), and season (Apr-Sept and Oct-Nov).

#### Effects of hunting

**Fig 4:**
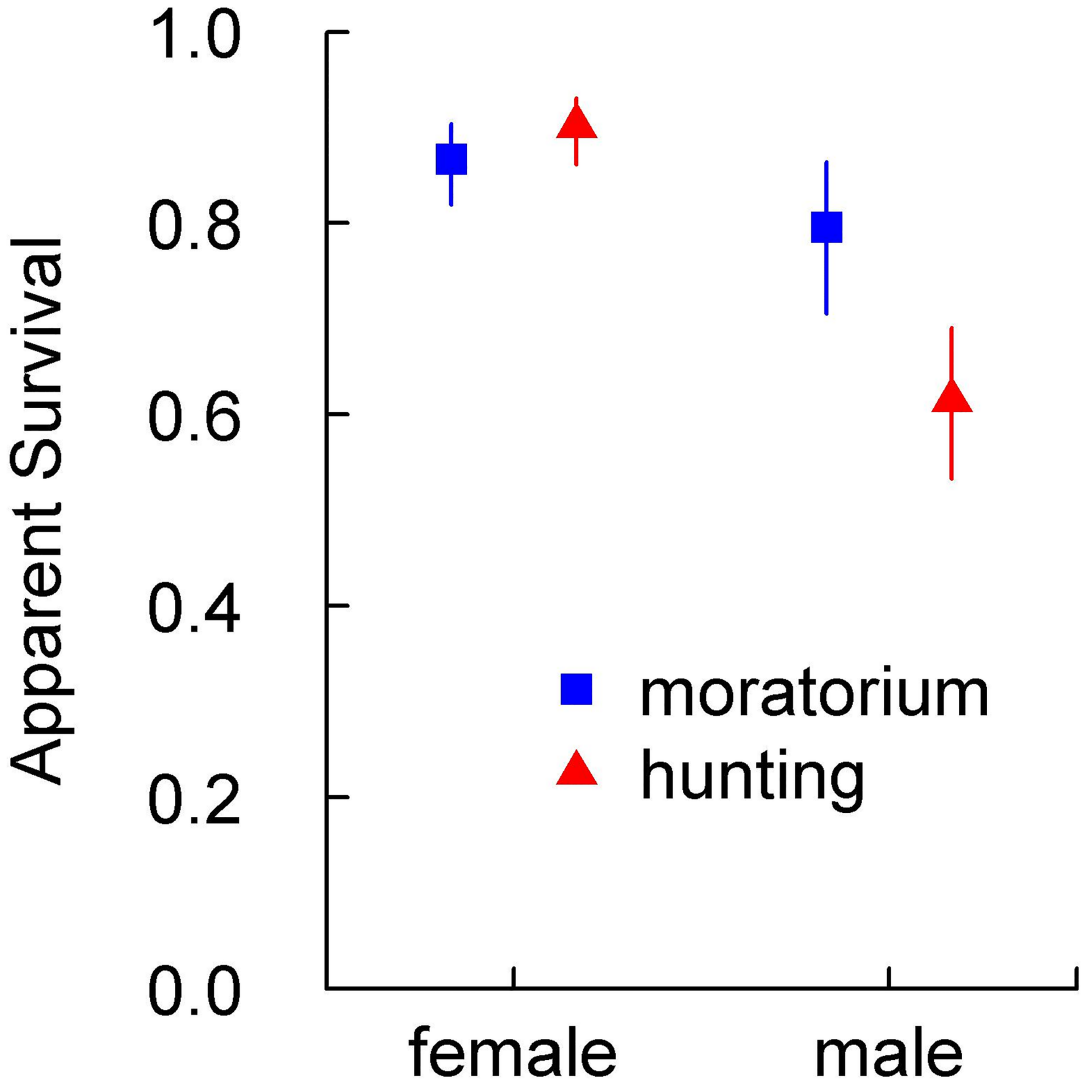
Apparent survival estimates in all lions ≥ 2 years (with 90% CI), during and before a moratorium on trophy hunting of adult male lions. Survival estimates are derived from a CJS model fit a posteriori including the same parameters effecting detection as in the top model. For adult males, survival rates significantly differ at the α = 0.075 level.

None of the 22 models without a hunting effect on mean annual lion survival were within 2 AICc units of the top model. All four of the top models included an increase in survival during the hunting moratorium for males ≥ 2 years (Table 1 and S2 Table). The top model included a significant negative effect of hunting on male survival (-0.765, 95% CL: -1.494, -0.037) that was comparable in magnitude to coefficients describing large age and sex effects on survival (S2 Table). Confidence intervals of expected survival derived from the top model overlapped (Fig 3) as they included the compounded uncertainty of age, sex, and hunting effects on survival (S2 Table). Expected mean annual apparent survival increased during the moratorium from 0.565 (0.446, 0.676) to 0.736 (0.580, 0.849) in subadult males and from 0.683 (95% CI: 0.539, 0.799) to 0.823 (0.704, 0.900) in adult males (Fig 3). The 4^th^ ranked model included a negative effect of hunting for all lions ≥2 years, not just male lions (Table 1). However, females generally showed high survival even during hunting, and translating the negative effect of hunting from the 4^th^ ranked model onto female survival translated into a decrease only 0.04, from 0.948 (0.906, 0.972) to 0.911 (0.860, 0.945). We fit one additional survival model, *a posteriori*, that excluded cubs and pooled all lions ≥ 2 years into a single age class, with a separate hunting effect term on each gender. This model was fit to expose any underlying variation between the response of male and female survival to the moratorium on hunting. Survival estimates derived from the *a posteriori* model showed no effect of hunting on females (coefficient: 0.343; 95% CL: -0.323, 1.008) and a strongly negative effect of hunting on males (coefficient: -0.871; 95% CL: -1.587, -0.156). The estimated 17.8 percentage point increase in ≥2-year-old male expected survival during the moratorium (Fig 4) was more precise than the difference in ≥4-year-old male survival estimated by the top model (Fig 3) because the hunting effect from the top model could be solely allocated to a single age class.

Top models varied in estimates of hunting effects on mean annual cub survival. Two of the four top models (including the best model) did not include any hunting effect on cub survival, but a positive effect was included in the other two models. Model coefficients from the 2^nd^ and 4^th^ ranked models (Table 1) were relatively small and imprecise (coefficient ± SE: 0.309 ± 0.341 and 0.286 ± 0.345, respectively). These coefficients translated into declines of 0.086 and 0.068 (respectively) in 1^st^ year cub survival and 0.035 and 0.034 (respectively) in 2^nd^ year cub survival during the moratorium.

### Abundance

The 8 annual abundance models produced 32 separate estimates of detection probability (2 seasons × 2 mixture class for each of 8 models of annual abundance, S3 Table), mirroring the parameterization of the detection process from the CJS survival model (2 seasons × 2 mixture classes × 8 years, S2 Table). After fixing the mixing parameter, π, at the model-average estimate from the top four survival models (0.523 ± 0.038 SE), model fitting algorithms converged for data from each year except for first year (2008). The interaction term between detection class and season was dropped from the 2008 abundance model before refitting, which allowed convergence.

**Fig 5:**
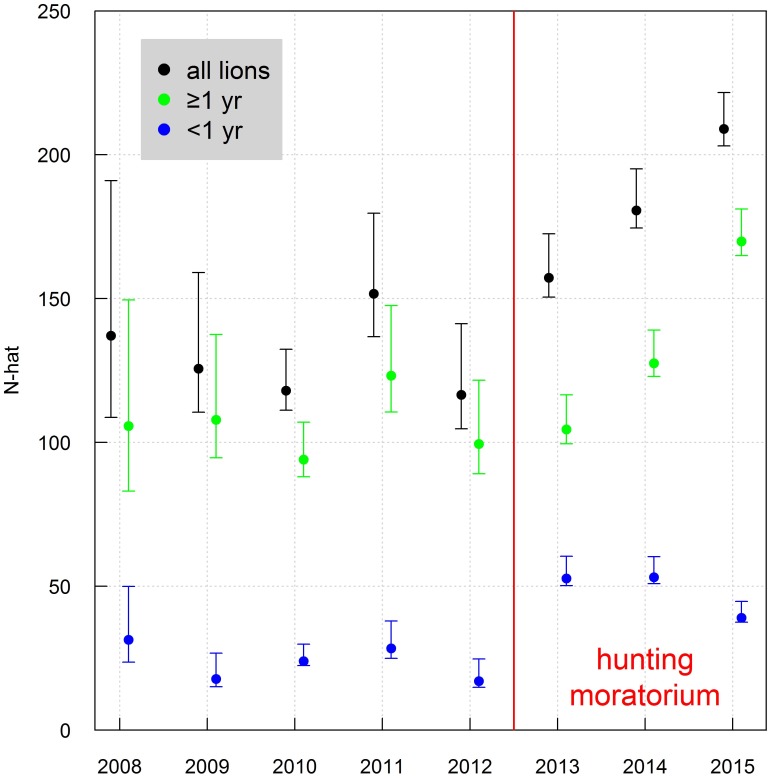
Estimation of the size (with upper and lower 95% CI) of the South Luangwa lion population from 2008 to 2015 before and during a 3-year moratorium on trophy hunting of male lions from eight closed population capture-recapture models accounting for imperfect detection arising from individual heterogeneity and temporal effects of season and study year.

Lion numbers increased from a low of 116 lions in 2012, just prior to the moratorium, to a high of 209 lions in 2015, the third (and last) year of the moratorium (Table 2). This increase was due to a combination of increased male survival and a large number of cubs produced in every year during the moratorium (Fig 5). The number of first-year cubs tripled in the first year of the moratorium (Table 2) followed by an increase in the number of lions ≥1 year in the 2^nd^ year, and an increase in the number of known subadults in the third year of the moratorium (Fig 2) indicating successful recruitment of cubs into older age classes. In short, with the implementation of the moratorium, high numbers of newborn cubs, followed by annual increases in the number of lions ≥1 year old resulted in a steady increase in abundance throughout the recovery period, with the largest number of known individuals (Table 2) and the largest population estimate over the 8-year period (Fig 5) occurring by the end of the three-year moratorium.

**Table 2:** Estimates of annual abundance of the South Luangwa lion population from 2008 to 2015.

year	known lions	known mortalities	N-hat					
			lions < 1 yr	lions ≥ 1 yr	All lions	SE	95% LCL	95% UCL
2008	76	1	31.5	105.7	137.2	20.1	108.7	191.0
2009	95	3	17.8	107.9	125.7	11.6	110.5	159.1
2010	92	13	24.0	94.1	118.0	5.1	111.2	132.4
2011	111	9	28.4	123.3	151.7	10.5	136.8	179.7
2012	88	6	17.1	99.5	116.5	8.8	104.7	141.3
2013	138	7	52.7	104.6	157.3	5.3	150.5	172.5
2014	170	0	53.1	127.5	180.6	4.9	174.5	195.1
2015	198	0	39.0	169.9	209.0	4.5	203.1	221.7

Estimates were obtained from eight closed mark-recapture models accounting for variation in detection arising from detection class (*p*_*high*_, p_low_) and season (cool dry, Apr–Sept and hot dry, Oct-Nov), {p(h_2_ × season),c(),pi(.)}.

## Discussion

In the five years preceding the hunting moratorium, Rosenblatt et al. [27] showed skewed demography and decreasing abundance in the SLNP lion population corresponding with 46 males that were harvested from the study area in that period. In accordance with the recommendation of Rosenblatt et al. [27], the Zambian Department of National Parks and Wildlife administered the hunting moratorium for three years to support a younger female age distribution, increased recruitment of cubs, and increased number of adult males [15]. While this policy was controversial, we can conclude the moratorium contributed to creating the desired changes in the lion population: (1) known and estimated first year cubs in the population are at the highest levels observed (2) the age distribution of adult females has shifted to a younger population, (3) there were 3 times more known adult males in the population in 2015 than in any of the five years preceding the moratorium, and (4) the number of males of harvestable age has increased. Our top model confirmed a negative effect of trophy hunting on subadult and adult male survival. Subadult male and adult male apparent survival estimates in the presence of trophy hunting were comparable to that seen in 1^st^ year cubs (Fig 3), an age class that shows poor survival in most populations [38–40]. Along with higher survival of subadult males during the three-year moratorium, we identified eight new adult males that had immigrated or previously gone undetected in our study area (in comparison to nine total males first identified as adults over the previous 5-year period). Models that allowed for difference in survival among adult male age classes were not supported and this could be due to the small number of adult male lions in our sample, particularly during trophy hunting (Fig 2). Because no adult male age class held more than a few individuals, additional years of data (or data from other populations) might support models with more complex age effects in adult male lion survival. For example, three years of data in addition to the 5 years used by Rosenblatt et al. [27] resolved a difference in survival between first-year and second-year cubs that was not previously detectable, and showed adult females lived to age 10 before survival declined sharply (Fig 3).

We also found evidence for a growing lion population during the moratorium due to a combination of improved adult male survival and increased cub recruitment. The age class with lowest apparent survival estimate was first-year cubs, but our estimate was based on detections that occurred after cubs averaged 4.4 months old. Even though we exponentiated the survival estimate for first year cubs, our estimate is probably biased high if cub survival over the first four months is lower than survival over the remainder of the first year. Our top four survival models included two models, neither of them the best model, with decreased cub survival during the hunting moratorium. The number of known and estimated cubs in the population was greater in all years of the moratorium (Figs 2 and 5, Table 1) and negative density dependent effects on cub survival [41–43] may have been acting more strongly during the moratorium. During moratorium years, we observed nearly twice as many known adult males as we did in any year prior to the moratorium, which may have increased the opportunity for infanticide [12, 29] and transitional dynamics as the male population and coalition structures and tenure changed rapidly (Fig 2). Despite the small and non-significant decline (based on overlapping confidence intervals) in cub survival during the moratorium, an increase in the total number of cubs was the primary driver of the increase in lion abundance during the moratorium (Table 1; Fig 5). The dramatic increase in male survival during the moratorium may have increased breeding male availability and pride male tenure favoring cub production. The population’s age-sex structure also changed substantially during the three-year recovery period, including an unexpected change in the sex-ratio of cubs (Fig 2), but note that sex-ratio in mammals can be affected by many factors including, importantly, parent age [44]. Whether these shifts represent transitional responses between the hunting and moratorium periods or whether they represent a new phase in lion demographics is not clear. Additional years of data may resolve these patterns.

Other factors might logically explain a change in lion survival and abundance, such as poaching for lion parts, snaring by-catch, changes in prey density, or disease, and these forces are at play in the South Luangwa lion population [10, 11, 27]. These factors do not explain the trend in abundance we observed, however. We observed most (55.4% to 94.7%) of the lions estimated to be in the population each year (Table 2) and simultaneously monitored mortalities (Table 2), snaring by-catch, and prey density. We observed 3.4 lions/year carrying snares during the hunting period and 3.7 lions/year during the moratorium. During the study, we confirmed only two mortalities that appeared to be caused by an injury from a wire snare, one during the hunting period and one during the moratorium. We documented two mortalities that appeared to be due to disease, one in each time period. Most causes of mortality could not be confirmed but starvation that might have indicated low prey availability was never suspected. Additionally, increases in elephant poaching—and the subsequent increase in carcasses as a lion food source—do not explain increases in lion numbers following cessation of trophy hunting. The SLNP lions did not utilize elephant carcasses as a significant food source and elephant poaching increases did not correlate with years of hunting/no-hunting [45]. Declines in disease, poaching, prey scarcity or other unmeasured environmental factors that may have coincided with the moratorium to explain increasing adult male survival would also be expected to affect survival of adult females and cubs, yet we observe only neutral or non-significant declines in survival in these age-sex classes. Movement seems unlikely to explain the patterns we observed because, Rosenblatt et al. [27] found the number of missing males (n = 46), and the timing of their disappearance closely matched the number and timing of harvested males (n = 46), suggesting emigration from the study area weakly affected our apparent survival estimates for male lions during trophy hunting. Lastly, immigration also seems unlikely to explain the increase in abundance because immigrating adults would be evident in our data as new (previously undetected) individuals in the subadult or adult age class, yet the number of new subadult and adults was often very small and highly comparable across years (Fig 2).

Because most lions in this study used both SLNP and GMAs and hunting was restricted to GMAs, the dramatic increase in male survival and lion abundance suggests strong evidence of a “vacuum effect”, wherein vacant breeding male opportunities in hunting areas attract males from protected areas [20]. Our results support previous conclusions that South Luangwa lions were not buffered from the impacts of trophy hunting due to these source-sink dynamics [10, 22, 27]. Male lions are drawn towards the GMAs to fill territorial vacancies created by trophy hunting (and sometimes by accidental wire-snare poaching [10]), where their survival is reduced in the presence of trophy hunting. The dynamics are more complex than simply a negative effect of hunting on the survival on males living outside or leaving a protected area. The large increase in cub numbers observed during the moratorium demonstrates how the effects of trophy hunting can propagate across protected area boundaries when animals can move freely, negatively impacting growth in lion populations that are a source for trophy-hunted males, the apex predator in a protected ecosystem, and an important draw for phototourism. Such widespread impacts emphasize that sustainable hunting of large carnivores is dependent on generally conservative harvest policies, particularly where steep gradients in protection exist.

Incentive-based quotas have been implemented inside reserves wherein the harvest of a suitably-aged male results in a quota increase for the following year, sometimes quite significantly [46]. However, reactive quotas still carry the risk the population can decline at a faster rate than the harvest can be adjusted, particularly when hunters increase their effort to confront scarcity [46]. Secondly, rewarding concessions with higher quotas after harvesting older male could reinforce vacuum effects in lion populations straddling strong gradients of protection. Finally, such policies may overestimate how well the demographic composition of harvest reflects the composition of the remaining population or its ability to sustain further offtake [47]. The stochastic nature of lion populations and the remaining uncertainty surrounding demography suggests that policies that reward long-term persistence over short-term gains may be more sustainable [15, 47]. For example, harvest policies that also consider prey density may better link lion offtake with production and would reward stakeholder engagement in larger conservation issues, such as poaching for the skin and bone trade and the growing illegal bushmeat trade [14] which directly and indirectly kills lions through by-catch and prey depletion [10].

## Conclusion

### Future management and conservation of lions

Populations of large carnivores worldwide are in decline and quantifying the anthropogenic impacts of factors like trophy hunting will continue to be important for the development of conservation strategies. Around the world, many conservation and natural resource agencies advocate using adaptive management when deciding how to conserve wildlife and ecosystems in the face of uncertainty. A foundational principle of adaptive management is ongoing monitoring of systems as regulations change to generate valuable insight into system functioning that can feedback into future policy formation [46]. Strong evidence that trophy hunting was driving population declines and skew in lion demography resulted from intensive monitoring of the South Luangwa lion population before and after the hunting moratorium. Moving forward, agencies that regulate trophy hunting may wish to avoid conditions that produced the patterns seen in the South Luangwa lion population prior to the moratorium, especially given that these patterns were apparent in a lion population using a large protected area. While the response in the lion population we observed was large, we caution against the use of regular hunting moratoria as a panacea. Any sustainable harvesting strategy must consist of conservative quotas, age-based harvesting, and systematic monitoring to address the inherent uncertainty that accompanies our understanding of demography in naturally sparse large carnivores [15]. Likewise, many factors limit lion populations and conservative harvest policies, including the use of hunting moratoria, may not address other limiting and likely additive factors (e.g., habitat loss, wire snare by-catch, prey-depletion, poaching, retaliatory killing, and disease) that threaten the persistence of lion populations across Africa.

## Supporting information

S1 Data Input data file. Final data file containing encounter histories for the period between 2008–2015.
(CSV)

S1 Table Models fit to describe age and sex-specific survival in the study lion population from 2008–2015. Along with a base model derived from Rosenblatt et al. (2014) that identified *cubs* (0.00–1.99 years), *subadults* (2.00–3.99 years), and 3 adult age categories: *young adults* (4.00–5.99 years), *prime adults* (6.00–7.99 years), and *old adults* (≥8.00 years), we considered 21 other variations in age-sex class structure potentially affecting survival. We fit each model below by collapsing the effect of the first term into the intercept and fit the remaining terms as adjustments to the intercept. All models were fit in a second stage of analysis using the detection parameters from the best model identified in the first stage of analysis. For each model listed, we also fit 12 variants that included the effects of hunting as additional additive terms to separate age-sex classes.
(DOCX)

S2 Table Best supported CJS model coefficient estimates. Coefficient estimates from the best supported Cormack-Jolly-Seber model of age, gender, and trophy hunting effects on lion survival and individual heterogeneity, season, and year effects on detection {Phi(age_[0,1,2,4+)_ & ♀_[2,4,10+)_ & hunting:♂_[2+)_), p(mixture*season*year), pi(.)}.
(DOCX)

S3 Table Estimated detection probability. Estimated detection probability from eight closed mark-recapture model of annual abundance assuming detection varied as a function of individual heterogeneity (p_low_ and p_high_) and season (cool dry, Apr–Sept and hot dry, Oct-Nov), {p(h_2_ × season),c(),pi(.)}. The mixing parameter (π) and its complement (1 –π) describing the probabilities that each lion belonged to the p_high_ and p_low_ detection classes, respectively, was fixed at 0.523 in all years. For 2008, the model did not converge on reasonable estimates until the interaction between season and mixture class was dropped from the model.
(DOCX)
