# Supplementary material for: Quantifying lion (Panthera leo) demographic response following a three-year moratorium on trophy hunting
Source: PLoS One. 2018 May 21;13(5):e0197030. doi: 10.1371/journal.pone.0197030 (PMC5962075; doi:10.1371/journal.pone.0197030)
Supplement: S1 Table — Along with a base model derived from Rosenblatt et al. (2014) that identified cubs (0.00–1.99 years), subadults (2.00–3.99 years), and 3 adult age categories: young adults (4.00–5.99 years), prime adults (6.00–7.99 years), and old adults (≥8.00 years), we considered 21 other variations in age-sex class structure potentially affecting survival. We fit each model below by collapsing the effect of the first term into the intercept and fit the remaining terms as adjustments to the intercept. All models were fit in a second stage of analysis using the detection parameters from the best model identified in the first stage of analysis. For each model listed, we also fit 12 variants that included the effects of hunting as additional additive terms to separate age-sex classes. (DOCX) [file pone.0197030.s002.docx]

| **S1 Table. Models fit to describe age and sex-specific survival in the study lion population from 2008-2015**. Along with a base model derived from Rosenblatt et al. (2014) that identified *cubs* (0.00-1.99 years), *subadults* (2.00-3.99 years), and 3 adult age categories: *young adults* (4.00-5.99 years), *prime adults* (6.00-7.99 years), and *old adults* (≥8.00 years), we considered 21 other variations in age-sex class structure potentially affecting survival. We fit each model below by collapsing the effect of the first term into the intercept and fit the remaining terms as adjustments to the intercept. All models were fit in a second stage of analysis using the detection parameters from the best model identified in the first stage of analysis. For each model listed, we also fit 12 variants that included the effects of hunting as additional additive terms to separate age-sex classes. | | |
| --- | --- | --- |
| # | Deviation from base model | Model Structure |
| 1 | BASE MODEL (derived from Rosenblatt et al 2014) | age_[0,2)_ + age_[2,4)_ + age_[4+)_ + ♀_[0,2)_ + ♀_[2,4)_ + ♀_[4,6)_ + ♀_[6,8)_ + ♀_[8+)_ |
| 2 | Φ varies within **old adult** females | age_[0,2)_ + age_[2,4)_ + age_[4+)_ + ♀_[0,2)_ + ♀_[2,4)_ + ♀_[4,6)_ + ♀_[6,8)_ + ♀_[8,10)_ + ♀_[10+)_ |
| 3 | Φ differs between 1^st^ and 2^nd^ year **cubs** | age_[0,1)_ + age_[1,2)_ + age_[2,4)_ + age_[4+)_ + ♀_[0,2)_ + ♀_[2,4)_ + ♀_[4,6)_ + ♀_[6,8)_ + ♀_[8+)_ |
| 4 | Φ differs between 1^st^ and 2^nd^ year **cubs**  Φ varies within **old adult** females | age_[0,1)_ + age_[1,2)_ + age_[2,4)_ + age_[4+)_ + ♀_[0,2)_ + ♀_[2,4)_ + ♀_[4,6)_ + ♀_[6,8)_ + ♀_[8,10)_ + ♀_[10+)_ |
| 5 | Φ differs between 1^st^ and 2^nd^ year **cubs**  Φ does not differ across gender in 1^st^ year **cubs**  Φ varies within **old adult** females | age_[0,1)_ + age_[1,2)_ + age_[2,4)_ + age_[4+)_ + ♀_[1,2)_ + ♀_[2,4)_ + ♀_[4,6)_ + ♀_[6,8)_ + ♀_[8,10)_ + ♀_[10+)_ |
| 6 | Φ differs between 1^st^ and 2^nd^ year **cubs**  Φ does not differ across gender in **cubs**  Φ varies within **old adult** females | age_[0,1)_ + age_[1,2)_ + age_[2,4)_ + age_[4+)_ + ♀_[2,4)_ + ♀_[4,6)_ + ♀_[6,8)_ + ♀_[8,10)_ + ♀_[10+)_ |
| 7 | Φ differs between 1^st^ and 2^nd^ year **cubs**  Φ does not differ across gender in 1^st^ year **cubs**  Φ constant in adult females  Φ changes between **young adult** and older adult males | age_[0,1)_ + age_[1,2)_ + age_[2,4)_ + age_[4+)_ + ♂_[1,2)_ + ♂_[2,4)_ + ♂_[4,6)_ + ♂_[6+)_ |
| 8 | Φ differs between 1^st^ and 2^nd^ year **cubs**  Φ does not differ across gender in **cubs**  Φ differs between **young adult** males and older males  Φ varies within **old adult** females | age_[0,1)_ + age_[1,2)_ + age_[2,4)_ + age_[4,6)_ + age_[6+)_ + ♀_[2,4)_ + ♀_[4,6)_ + ♀_[6,8)_ + ♀_[8,10)_ + ♀_[10+)_ |
| 9 | Φ differs between 1^st^ and 2^nd^ year **cubs**  Φ does not differ across gender in 1^st^ year **cubs**  Φ differs between **young adult** males and older males  Φ varies within **old adult** females | age_[0,1)_ + age_[1,2)_ + age_[2,4)_ + age_[4,6)_ + age_[6+)_ + ♀_[1,2)_ + ♀_[2,4)_ + ♀_[4,6)_ + ♀_[6,8)_ + ♀_[8,10)_ + ♀_[10+)_ |
| 10 | Φ differs between 1^st^ and 2^nd^ year **cubs**  Φ differs between **young adult** males and older males  Φ varies within **old adult** females | age_[0,1)_ + age_[1,2)_ + age_[2,4)_ + age_[4,6)_ + age_[6+)_ + ♀_[0,2)_ + ♀_[2,4)_ + ♀_[4,6)_ + ♀_[6,8)_ + ♀_[8,10)_ + ♀_[10+)_ |
| 11 | Φ differs between 1^st^ and 2^nd^ year **cubs**  Φ differs between **young adult** males and older males  Φ varies within **old adult** females | age_[0,1)_ + age_[1,2)_ + age_[2,4)_ + age_[4,6)_ + age_[6+)_ + ♀_[0,2)_ + ♀_[2,4)_ + ♀_[4,6)_ + ♀_[6,10)_ + ♀_[10+)_ |
| 12 | Φ differs between 1^st^ and 2^nd^ year **cubs**  Φ constant from 6-10 in adult females | age_[0,1)_ + age_[1,2)_ + age_[2,4)_ + age_[4+)_ + ♀_[0,2)_ + ♀_[2,4)_ + ♀_[4,6)_ + ♀_[6,10)_ + ♀_[10+)_ |
| 13 | Φ differs between 1^st^ and 2^nd^ year **cubs**  Φ differs for **old adult** males  Φ varies within **old adult** females | age_[0,1)_ + age_[1,2)_ + age_[2,4)_ + age_[4,8)_ + age_[8+)_ + ♀_[0,2)_ + ♀_[2,4)_ + ♀_[4,6)_ + ♀_[6,8)_ + ♀_[8,10)_ + ♀_[10+)_ |
| 14 | Φ differs between 1^st^ and 2^nd^ year **cubs**  Φ does not differ across gender in 1^st^ year **cubs**  Φ changes in adult males after age 8  Φ varies within **old adult** females | age_[0,1)_ + age_[1,2)_ + age_[2,4)_ + age_[4,8)_ + age_[8+)_ + ♀_[1,2)_ + ♀_[2,4)_ + ♀_[4,6)_ + ♀_[6,8)_ + ♀_[8,10)_ + ♀_[10+)_ |
| 15 | Φ differs between 1^st^ and 2^nd^ year **cubs**  Φ does not differ across gender in **cubs**  Φ changes in adult males after age 8  Φ varies within **old adult** females | age_[0,1)_ + age_[1,2)_ + age_[2,4)_ + age_[4,8)_ + age_[8+)_ + ♀_[2,4)_ + ♀_[4,6)_ + ♀_[6,8)_ + ♀_[8,10)_ + ♀_[10+)_ |
| 16 | Φ differs between 1^st^ and 2^nd^ year **cubs**  Φ does not differ across gender in **cubs**  Φ changes in adult males after age 8 | age_[0,1)_ + age_[1,2)_ + age_[2,4)_ + age_[4,8)_ + age_[8+)_ + ♀_[2,4)_ + ♀_[4,6)_ + ♀_[6,8)_ + ♀_[8+)_ |
| 17 | Φ differs between 1^st^ and 2^nd^ year **cubs**  Φ does not differ across gender in 1^st^ year **cubs**  Φ changes in adult males after age 8 | age_[0,1)_ + age_[1,2)_ + age_[2,4)_ + age_[4,8)_ + age_[8+)_ + ♀_[1,2)_ + ♀_[2,4)_ + ♀_[4,6)_ + ♀_[6,8)_ + ♀_[8+)_ |
| 18 | Φ differs between 1^st^ and 2^nd^ year **cubs**  Φ changes in adult males after age 8 | age_[0,1)_ + age_[1,2)_ + age_[2,4)_ + age_[4,8)_ + age_[8+)_ + ♀_[0,2)_ + ♀_[2,4)_ + ♀_[4,6)_ + ♀_[6,8)_ + ♀_[8+)_ |
| 19 | Φ differs between 1^st^ and 2^nd^ year **cubs**  Φ does not differ across gender in **cubs**  Φ in adult females is constant to age 10 | age_[0,1)_ + age_[1,2)_ + age_[2,4)_ + age_[4+)_ + ♀_[2,4)_ + ♀_[4,10)_ + ♀_[10+)_ |
| 20 | Φ differs between 1^st^ and 2^nd^ year **cubs**  Φ in adult females is constant to age 10 | age_[0,1)_ + age_[1,2)_ + age_[2,4)_ + age_[4+)_ + ♀_[0,2)_ + ♀_[2,4)_ + ♀_[4,10)_ + ♀_[10+)_ |
| 21 | Φ differs between 1^st^ and 2^nd^ year **cubs**  Φ does not vary between male and female **cubs**  Φ in adult females is constant to age 8  Φ varies within **old adult** females | age_[0,1)_ + age_[1,2)_ + age_[2,4)_ + age_[4+)_ + ♀_[2,4)_ + ♀_[4,8)_ + ♀_[8,10)_ ♀_[10+)_ |
| 22 | Φ differs between 1^st^ and 2^nd^ year **cubs**  Φ does not differ across gender in **cubs**  Φ is constant between **young adult** and **prime adult** females | age_[0,1)_ + age_[1,2)_ + age_[2,4)_ + age_[4+)_ + ♀_[2,4)_ + ♀_[4,8)_ + ♀_[8+)_ |
|  | | |
