# Supplementary material for: Quantifying lion (Panthera leo) demographic response following a three-year moratorium on trophy hunting
Source: PLoS One. 2018 May 21;13(5):e0197030. doi: 10.1371/journal.pone.0197030 (PMC5962075; doi:10.1371/journal.pone.0197030)
Supplement: S2 Table — Coefficient estimates from the best supported Cormack-Jolly-Seber model of age, gender, and trophy hunting effects on lion survival and individual heterogeneity, season, and year effects on detection {Phi(age[0,1,2,4+) & ♀[2,4,10+) & hunting:♂[2+)), p(mixture*season*year), pi(.)}. (DOCX) [file pone.0197030.s003.docx]

| **S2 Table. Best supported CJS model coefficient estimates.** Coefficient estimates from the best supported Cormack-Jolly-Seber model of age, gender, and trophy hunting effects on lion survival and individual heterogeneity, season, and year effects on detection {Phi(age_[0,1,2,4+)_ & ♀_[2,4,10+)_ & hunting:♂_[2+)_), p(mixture*season*year), pi(.)}. | | | | | |
| --- | --- | --- | --- | --- | --- |
| Parameter | Effect | β | SE | 95% LCL | 95% UCL |
| pi | Intercept | 0.094 | 0.151 | -0.202 | 0.390 |
| Phi | Intercept (0-1 cub survival) | 0.632 | 0.183 | 0.273 | 0.991 |
| Phi | (1,2) age adjustment | 1.313 | 0.446 | 0.439 | 2.187 |
| Phi | [2,4) age adjustment | 0.393 | 0.402 | -0.396 | 1.182 |
| Phi | [4+) age adjustment | 0.901 | 0.386 | 0.146 | 1.657 |
| Phi | female [2,4) adjustment | 1.028 | 0.499 | 0.050 | 2.005 |
| Phi | female [4,10) adjustment | 0.993 | 0.418 | 0.174 | 1.812 |
| Phi | female[10+) adjustment | -0.953 | 0.452 | -1.840 | -0.067 |
| Phi | hunted male [2+) adjustment | -0.765 | 0.372 | -1.494 | -0.037 |
| p | Intercept | -0.245 | 0.563 | -1.347 | 0.858 |
| p | p_low_ mixture class adjustment | 0.929 | 1.147 | -1.319 | 3.177 |
| p | hot dry season adjustment | -0.801 | 0.739 | -2.250 | 0.647 |
| p | year 2 adjustment | 1.121 | 0.614 | -0.084 | 2.325 |
| p | year 3 adjustment | 0.763 | 0.597 | -0.407 | 1.933 |
| p | year 4 adjustment | 0.853 | 0.603 | -0.329 | 2.036 |
| p | year 5 adjustment | 0.227 | 0.612 | -0.972 | 1.425 |
| p | year 6 adjustment | 1.731 | 0.614 | 0.528 | 2.935 |
| p | year 7 adjustment | 2.454 | 0.623 | 1.232 | 3.676 |
| p | year 8 adjustment | 1.336 | 0.586 | 0.187 | 2.485 |
| p | p_low_ × hot dry season adjustment | -2.493 | 1.504 | -5.441 | 0.454 |
| p | p_low_ × year 2 adjustment | -3.134 | 1.118 | -5.326 | -0.943 |
| p | p_low_ × year 3 adjustment | -3.403 | 1.138 | -5.633 | -1.173 |
| p | p_low_ × year 4 adjustment | -3.102 | 1.196 | -5.446 | -0.759 |
| p | p_low_ × year 5 adjustment | -3.905 | 1.226 | -6.307 | -1.502 |
| p | p_low_ × year 6 adjustment | -3.346 | 1.188 | -5.675 | -1.017 |
| p | p_low_ × year 7 adjustment | -3.593 | 1.191 | -5.928 | -1.259 |
| p | p_low_ × year 8 adjustment | -2.103 | 1.169 | -4.395 | 0.189 |
| p | hot dry season × year 2 adjustment | 0.260 | 0.794 | -1.297 | 1.817 |
| p | hot dry season × year 3 adjustment | 0.688 | 0.809 | -0.897 | 2.273 |
| p | hot dry season × year 4 adjustment | 0.894 | 0.827 | -0.726 | 2.514 |
| p | hot dry season × year 5 adjustment | 2.611 | 0.910 | 0.828 | 4.394 |
| p | hot dry season × year 6 adjustment | 3.318 | 1.262 | 0.844 | 5.792 |
| p | hot dry season × year 7 adjustment | 2.813 | 1.279 | 0.306 | 5.319 |
| p | hot dry season × year 8 adjustment | 1.028 | 0.804 | -0.549 | 2.605 |
| p | p_low_ × hot dry season × year 2 adjustment | 2.177 | 1.611 | -0.982 | 5.335 |
| p | p_low_ × hot dry season × year 3 adjustment | 4.107 | 1.548 | 1.073 | 7.141 |
| p | p_low_ × hot dry season × year 4 adjustment | 2.851 | 1.593 | -0.272 | 5.974 |
| p | p_low_ × hot dry season × year 5 adjustment | 2.528 | 1.677 | -0.760 | 5.816 |
| p | p_low_ × hot dry season × year 6 adjustment | 0.502 | 1.846 | -3.115 | 4.120 |
| p | p_low_ × hot dry season × year 7 adjustment | 0.736 | 1.863 | -2.916 | 4.388 |
| p | p_low_ × hot dry season × year 8 adjustment | 2.290 | 1.576 | -0.799 | 5.379 |
