# Supplementary material for: Quantifying lion (Panthera leo) demographic response following a three-year moratorium on trophy hunting
Source: PLoS One. 2018 May 21;13(5):e0197030. doi: 10.1371/journal.pone.0197030 (PMC5962075; doi:10.1371/journal.pone.0197030)
Supplement: S3 Table — Estimated detection probability from eight closed mark-recapture model of annual abundance assuming detection varied as a function of individual heterogeneity (plow and phigh) and season (cool dry, Apr–Sept and hot dry, Oct-Nov), {p(h2 × season),c(),pi(.)}. The mixing parameter (π) and its complement (1 –π) describing the probabilities that each lion belonged to the phigh and plow detection classes, respectively, was fixed at 0.523 in all years. For 2008, the model did not converge on reasonable estimates until the interaction between season and mixture class was dropped from the model. (DOCX) [file pone.0197030.s004.docx]

| **S3 Table. Estimated detection probability.** Estimated detection probability from eight closed mark-recapture model of annual abundance assuming detection varied as a function of individual heterogeneity (p_low_ and p_high_) and season (cool dry, Apr – Sept and hot dry, Oct-Nov), {p(h_2_ × season),c(),pi(.)}. The mixing parameter (π) and its complement (1 – π) describing the probabilities that each lion belonged to the p_high_ and p_low_ detection classes, respectively, was fixed at 0.523 in all years. For 2008, the model did not converge on reasonable estimates until the interaction between season and mixture class was dropped from the model. | | | | | |
| --- | --- | --- | --- | --- | --- |
| Real parameter | p | SE | lower 95% CL | upper 95% CL |  |
| 2008 (m = 76) |  |  |  |  |  |
| p_high_, cool | 0.175 | 0.028 | 0.126 | 0.237 |  |
| p_high_, hot | 0.231 | 0.043 | 0.157 | 0.326 |  |
| p_low_, cool | 0.044 | 0.017 | 0.020 | 0.094 |  |
| p_low_, hot | 0.061 | 0.025 | 0.027 | 0.133 |  |
| 2009 (m = 95) |  |  |  |  |  |
| p_high_, cool | 0.443 | 0.037 | 0.371 | 0.517 |  |
| p_high_, hot | 0.370 | 0.060 | 0.262 | 0.493 |  |
| p_low_, cool | 0.083 | 0.036 | 0.034 | 0.185 |  |
| p_low_, hot | 0.122 | 0.043 | 0.060 | 0.233 |  |
| 2010 (m = 92) |  |  |  |  |  |
| p_high_, cool | 0.601 | 0.048 | 0.504 | 0.690 |  |
| p_high_, hot | 0.416 | 0.063 | 0.299 | 0.542 |  |
| p_low_, cool | 0.071 | 0.020 | 0.040 | 0.122 |  |
| p_low_, hot | 0.366 | 0.055 | 0.266 | 0.480 |  |
| 2011 (m = 111) |  |  |  |  |  |
| p_high_, cool | 0.401 | 0.037 | 0.330 | 0.476 |  |
| p_high_, hot | 0.448 | 0.058 | 0.339 | 0.562 |  |
| p_low_, cool | 0.082 | 0.022 | 0.048 | 0.138 |  |
| p_low_, hot | 0.133 | 0.043 | 0.069 | 0.242 |  |
| 2012 (m = 88) |  |  |  |  |  |
| p_high_, cool | 0.321 | 0.039 | 0.249 | 0.402 |  |
| p_high_, hot | 0.506 | 0.060 | 0.390 | 0.622 |  |
| p_low_, cool | 0.012 | 0.013 | 0.001 | 0.102 |  |
| p_low_, hot | 0.344 | 0.071 | 0.220 | 0.494 |  |
| 2013 (m = 138) |  |  |  |  |  |
| p_high_, cool | 0.506 | 0.032 | 0.443 | 0.568 |  |
| p_high_, hot | 0.785 | 0.053 | 0.664 | 0.870 |  |
| p_low_, cool | 0.174 | 0.029 | 0.124 | 0.239 |  |
| p_low_, hot | 0.267 | 0.047 | 0.184 | 0.369 |  |
| 2014 (m = 170) |  |  |  |  |  |
| p_high_, cool | 0.724 | 0.026 | 0.670 | 0.773 |  |
| p_high_, hot | 0.871 | 0.034 | 0.790 | 0.924 |  |
| p_low_, cool | 0.207 | 0.030 | 0.154 | 0.272 |  |
| p_low_, hot | 0.296 | 0.046 | 0.214 | 0.393 |  |
| 2015 (m = 198) |  |  |  |  |  |
| p_high_, cool | 0.569 | 0.027 | 0.516 | 0.621 |  |
| p_high_, hot | 0.469 | 0.042 | 0.389 | 0.550 |  |
| p_low_, cool | 0.214 | 0.025 | 0.168 | 0.268 |  |
| p_low_, hot | 0.324 | 0.043 | 0.246 | 0.412 |  |
